# An ab initio molecular dynamics investigation of the behaviour of amorphous substances in anodic aluminium oxide under electric field

**DOI:** 10.1038/s41598-024-58975-y

**Published:** 2024-05-07

**Authors:** Zeyu An, Shiyang Sun, Binghai Dong

**Affiliations:** 1 School of Materials Science and Engineering, Hubei University, Wuhan, Hubei 430000 People’s Republic of China; 2 School of Mechanical Engineering, Inner Mongolia University of Science & Technology, Baotou, Inner Mongolia 014010 People’s Republic of China

**Keywords:** Density functional theory, Molecular dynamics, Chemical physics, Two-dimensional materials

## Abstract

In order to elucidate the diffusion behaviour of ions in alumina during the anodic alumina process, the effects of electric field strength, hydration content, and electrolyte on amorphous alumina and hydrated alumina were studied using ab initio molecular dynamics. The results show that the diffusion rate of ions in alumina increases with the increase in electric field strength, but there is an extreme value. The maximum diffusion rate of Al ions in alumina monohydrate is 21.8 μm^2^/ms/V, while in alumina trihydrate, it is 16.7 μm^2^/ms/V. The ionic diffusion rate of hydrated alumina is one to two orders of magnitude larger than that of anhydrous amorphous alumina due to the effect of the drag of H ions, which reduces the migration activation energy. Electrolytes also affect the diffusion rate of alumina through the action of H ions. The increase in H ions will not only enhance the diffusion rate of hydrated alumina but also render the hydrous compound more vulnerable to breakdown.

## Introduction

**Figure 1:**
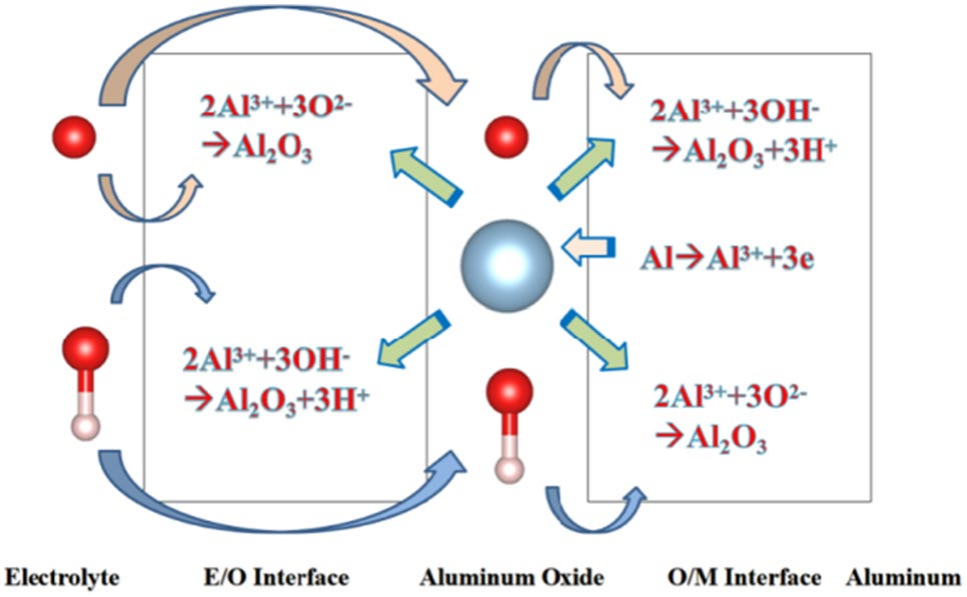
Schematic diagram showing the electrochemical reactions and ionic paths occurring during aluminium anodizing.

Since the beginning of the twentieth century, anodic aluminium oxide (AAO) has been widely used in industry for several applications, such as corrosion protection, colouring, and production of capacitors etc., due to its highly ordered arrangement, stable physical and chemical properties, and low cost^1,2^. Several experimental studies^3^ had investigated the effect of the anodizing conditions, especially the chemical composition of the electrolyte, voltage or current density, on most structural parameters of AAO, such as nanopore diameter, wall thickness. Several models, such as the field-assisted model^4,5^, the stress-induced plastic flow model^6,7^, the volume expansion stress model^8,9^ and the oxygen bubble model^10–12^, were proposed to elaborate the growth mechanism of the porous layers during anodizing. Although these theories have different principles, they are all based on a common cognition: the formation of AAO is a competition between the two reactions of aluminium oxidation and alumina dissolution. AAO grows stably when the reaction rates of these two are in equilibrium. Figure 1 shows the electrochemical reactions and ion diffusion occur during the aluminium anodizing. This complex electrochemical process involves three phases: metal aluminium, alumina, electrolyte and two interfaces: metal/oxide interface (M/O) and oxide/electrolyte interface (O/E).

In the competition between these two reactions, ion diffusion is the key factor determining whether a dense oxide film or a porous oxide film is present^2^. The dissolution reaction occurs at the O/E interface, and acid-induced dissolution plays a central role in this process^2^. The dissolution rate is determined by the speed of H+ crossing oxides. The growth rate of alumina depends on the ion diffusion rate of anions and cations, as shown in Fig. S1. Different ion diffusion rates determine the position where alumina forms. For general valve metals, when the metal ion diffusion rate is faster, oxides are generated near the metals. On the contrary, the oxide is generated near the electrolyte. For the formation process of AAO, the diffusion rates of the two ions may not differ significantly because the oxide is formed in the intermediate region. However, the oxide layer of aluminium is not a single component^13–16^. The specific formation position of the oxide film requires further study. Furthermore, to the best of the author’s knowledge, the main factors influencing the diffusion rate of ions in oxides and the relevant laws have not been addressed in existing research.

Murphy et al.^13^ proposed the Murphy model to describe the three-layer structure of the aluminium oxide film: the innermost layer was an anhydrous barrier oxide layer with a dense structure; the middle layer was a colloid layer, which was composed of hydrous oxides contaminated with low water content; the outer layer was a loose oxide with high water content and contained electrolyte anions. Dorsey also found that AAO has a hydrate grid and pointed out that the grid has a layered structure^14^. Subsequently, further research by Thompson demonstrated that ion migration causes alumina to become contaminated, forming a contaminated layer, as shown through the use of the isotope O18 tracer technique^15,16^. The experimental results confirmed that the alumina in AAO has an amorphous structure^9^. The above studies show that the oxide layer is composed of amorphous alumina, and the water content decreases with the increase in distance from the electrolyte.

Therefore, in the process of alumina formation and growth, it is not only necessary to explore the ion diffusion rate, including cations (Al^3+^, H^+^), anions (O^2−^, OH^−^), but also to consider the influence of different hydration states of the amorphous oxide. In this paper, to elucidate the behaviour of ions in an amorphous structure during the anodic alumina process, the first-principles molecular dynamics method was employed to investigate the impact of electric field strength, oxidation composition, and electrolyte environment on ion diffusion.

## Calculation method and details

The Vienna ab-initio simulation package (VASP)^17^, which is based on the pseudopotential plane wave approach and density functional theory, was used to conduct the simulations. The electron projector-augmented wave method^18^ was used to predict the interactions between ions and valence electrons. The Perdew-Burke-Ernzerhof generalized gradient approximation^19^ was used to determine the exchange–correlation function. The electronic occupancy was determined using the Methfessel-Paxton method^20^, with a smearing width of 0.05 eV. All self-consistent loops were iterated until the total energy difference among the systems, measured between adjacent iterating steps, reached less than 1 × 10^−5^ eV. The supercell was utilized to extend the wave functions with a kinetic energy cutoff of 400 eV.

Gutiérrez used molecular dynamics to study amorphous alumina^21^ and obtained a model consistent with the experiment conducted by Lamparter et al.^22^. Subsequently, he calculated the structural, elastic, vibrational, and electronic properties of amorphous alumina^23^. Based on the method of Gutiérrez et al., the paper utilized ab initio molecular dynamics (AIMD) to characterize anhydrous amorphous alumina, amorphous hydrated alumina, and selected alumina monohydrate and amorphous alumina trihydrate to represent water-poor and water-rich oxides, respectively. The amorphous cell (AC) module of the Accelrys Materials Studio (MS) package was used to construct the initial simulation box^24^. This method was used in a previous paper to establish the initial model^25^. To maintain the stoichiometric ratio, the simulation boxes contain 32 Al and 48 O atoms for amorphous alumina, 20 Al, 40 O, and 20 H atoms for amorphous alumina monohydrate, and 20 Al, 60 O, and 60 H atoms for amorphous alumina trihydrate. The AC module of the MS package uses the Monte Carlo method to establish the initial structure. The molecular dynamics model adopted a cubic supercell containing 80 atoms, with a volume of 9.46 × 9.46 × 9.46 Å^3^. The density of amorphous alumina represented by the model was 3.175 g/cm^3^. As for the other materials, the volume of supercells was 8.7245 × 8.7245 × 8.7245 Å^3^ for amorphous alumina monohydrate, which included 80 atoms, and for amorphous alumina trihydrate, which included 140 atoms, the volume was 9.089 × 9.089 × 9.089 Å^3^. The density was 3.40 g/cm^3^ for amorphous alumina monohydrate and 2.40 g/cm^3^ for amorphous alumina trihydrate. By using the AC module, 1000 structures were created. And the structure with the lowest total energy was selected.

Obviously, the structure obtained this way will have an unreasonable construction, even though the structure has been automatically optimized by the AC module to avoid issues such as vacuum regions and atomic positions being too close. Hence, the AIMD method was utilized for structural relaxation. Thus, all models were annealed from 700 to 300 K in 8 ps to ensure the amorphous structure of alumina, simulating a cooling rate of 0.05 K/fs to optimize the initial position of atoms set by the Monte Carlo method. The simulation consisted of a heating and holding process.

The modules were heated from 300 to 5000 K and then rapidly cooled to 500 K to ensure the alumina remained in an amorphous structure within 200 ps and 10 ps, respectively. The purpose of this step is to simulate quenching which using a fast-cooling rate in reality. The holding process was performed at 500 K for 10 picoseconds. In consideration of the significant heat generated locally during the oxidation reaction, the simulation temperature was set at 500 K. All diffusivity rate data were acquired during the holding process. The canonical ensemble (NVT) was used in all processes, and the temperature was controlled by the Nose–Hoover thermostat^26,27^. The simulation step was 1 femtosecond.

**Figure 2:**
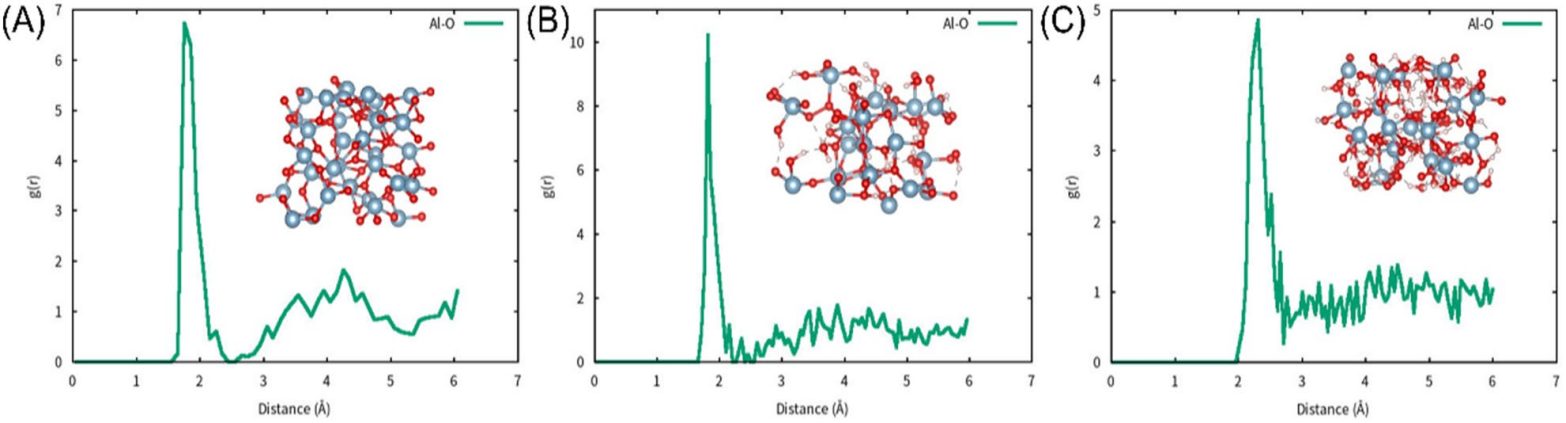
The RDF of Al-O bond in three models. The illustration in the figure is the corresponding structure. (**A**) amorphous alumina, (**B**) amorphous alumina monohydrate, (**C**) amorphous alumina trihydrate.

The radial distribution function (RDF) is an important equation for measuring crystal structure. It represents the probability of atoms appearing around an atom within a certain range. Figure 2 shows the RDF of all three alumina modules above, which exhibit amorphous structures. All the RDF data are based on the final modules at 500 K, which were used in the following paper. According to Fig. 2A, the bond length of Al-O is around 1.8 Å, and the radial distribution function (RDF) is approximately consistent with that reported in the literature^21,22^. In a supercell with periodic boundary conditions, an external electric field in the z-axis direction is allowed by including dipole correction in the vacuum region. The vacuum layer is 10 Å^28^, using the same method as described in previously published literature^29^. All the data obtained by analysing the AIMD results were processed using the VASPKIT package^30^.

Ionic diffusion coefficient (IDC) is closely related to mean square displacement (MSD). MSD refers to the displacement of an atom relative to its initial position at a specific moment, and its formula is:

$$ MSD = \left\langle {\left| {r\left( t \right) - r\left( 0 \right)} \right|^{2} } \right\rangle $$

where *r* represents the position at a certain moment, and <  > represents the calculation for all atoms in the group.

The IDC can be simply considered as a function of the slope of MSD, and the greater the slope of MSD, the greater the IDC. The specific relationship between the two can be quantified in the following formula within the cubic crystal system.

$$ IDC = \mathop {\lim }\limits_{t \to \infty } \frac{1}{6t}\left\langle {\left| {r\left( t \right) - r\left( 0 \right)} \right|^{2} } \right\rangle $$

## Results and discussion

### Influence of electric field strength

**Figure 3:**
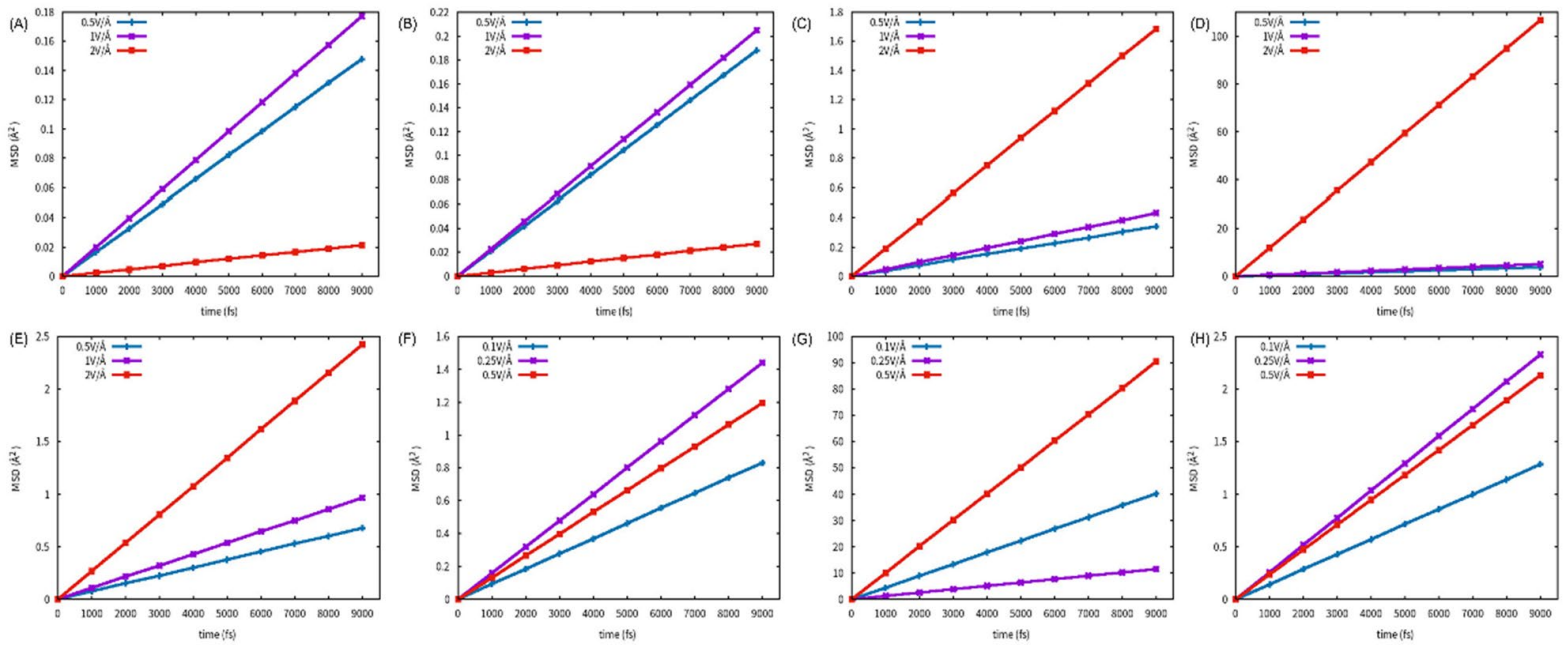
The MSD of three modules. (**A**, **B**) Al and O (anhydrous amorphous alumina), (**C–E**) Al, H, O (alumina monohydrate), (**F**–**H**) Al, H, O (alumina trihydrate).

Figure 3A, B displays the mean square displacement (MSD) of amorphous alumina at different electric field strengths. The IDC of Al and O increases and then decreases rapidly as the electric field strength rises from 0.5 to 2 V/Å, reaching its maximum at 1 V. Table S1 displays the correlation between electric field strength and IDC (ion diffusion coefficient) and ion mobility. To aid comprehension, we have included Fig. S5. The data suggests a non-linear relationship between electric field strength and IDC, with a maximum value of ion diffusion rate as the electric field strength increases. Additionally, when the electric field strength was too high and the anhydrous amorphous alumina had not been broken down, the IDC was low, indicating insulating properties. Under the aforementioned field strength, the IDCs of anhydrous amorphous alumina were very small due to the extreme stability of the Al–O bond. The stability of the Al–O bond makes it difficult for ions to move even in an electric field. This claim is supported by the slight structural change of the amorphous alumina over time in Fig. S2.

In alumina monohydrate, the mean square displacement (MSD) slope increases monotonically as the field strength increases, as shown in Fig. 3C–E. The MSD did not change significantly at field strengths of 0.5 V/Å and 1 V/Å, but only when the field strength reached 2 V/Å did the slope of the MSD line increase significantly. This suggests the presence of a specific energy barrier for ion diffusion in alumina monohydrate. When the electric field strength is low, the energy barrier prevents significant ion diffusion. Only when the electric field strength is sufficient to overcome the diffusion energy barrier will the IDC be significantly enhanced. Figure S3 displays the structural changes of alumina monohydrate over time under a 2 V/Å electric field. Under the influence of an electric field, H ions quickly dissociate from the constraint. Some of the aluminium ions became isolated hydrogen ions and migrated freely in alumina, while others remained bonded with oxygen ions and migrated together. Table S1 and the inserted image in Fig. S5 show that the ionic diffusion coefficient (IDC) of aluminium and oxygen in alumina monohydrate are similar, while the IDC of hydrogen is one order of magnitude higher than that of other ions. This indicates that, except for the isolated hydrogen ion, the hydrogen ions of the bonded oxygen ions induce and promote the migration of the oxygen ions. In a separate study, it was confirmed that H ions reduce the activation energy needed for O ion migration in aluminium crystals^31^.

The IDC of alumina trihydrate increased gradually with the enhancement of the electric field strength (Fig. 3F–H). Furthermore, under the influence of an electric field, alumina trihydrate dissociated more H ions and bonded H–O ions, resulting in a higher IDC. Table S1 shows that when the electric field strength is 0.5 V/Å, the IDC of alumina trihydrate is one order of magnitude higher than that of alumina monohydrate (0.20 vs 0.0629). This is also illustrated in Fig. S5. However, it is important to note that the enhancement effect is not infinite. The experiment^32^ showed a breakdown voltage, suggesting that in the oxidation process of AAO formation, the electric field strength does not need to be maximised; rather, there seems to be a threshold. Our simulation also confirmed that if the field strength is too strong, the alumina trihydrate will break down and be unable to maintain a stable structure. The electric field strength was adjusted to 0.1 V/Å, 0.25 V/Å, and 0.5 V/Å. Figure S5 displays that the IDC reaches its peak at an electric field strength of 0.25 V/Å. As the electric field is increased to 0.5 V/Å, the IDC starts to decline. This is due to the excessive migration of H ions, which partially offsets the driving force of the electric field on Al and O ions.

The growth rate of AAO film is typically proportional to the size of the IDC. Scholars have extensively studied the relationship between growth rate and voltage^2,32–34^. For porous AAO, the growth rate increases with higher voltage, which is directly related to electric field strength. Previous experiments have maintained a consistent experimental system, with the exception of varying voltage magnitude^33,34^. An increase in voltage can be interpreted as an increase in electric field strength. The conclusions regarding trends in this chapter align with those reported in previous experiments.

### Influence of oxide composition

The AAO oxidation process oxide layer is not a single component. It can be considered a hydrated oxide that varies along a gradient of water content between the metal and electrolyte, based on Thompson's model^15,16^. The composition of hydrated oxides has a significant impact on ion diffusion.

Figure 3 shows that the ionic conductivity of anhydrous amorphous alumina remains low even at high voltage, while that of alumina monohydrate is significantly higher. Its peak value is nearly 100 times greater than that of anhydrous alumina.

The evolution of the oxide structure in Fig. S3 suggests that the dissociated hydrogen ions in the hydrate contribute significantly to the conduction of an electric field. The H ion, being the smallest element, can easily migrate in the oxide under the influence of an electric field. As a result, the diffusion rate of the H ion is considerably higher than that of other ions, as shown in Table S1 and the purple and brown lines in Fig. S5. Moreover, some H ions have bonded with O, which can trigger and ‘drag’ the migration of O ions. And these H ions can also reduce the migration energy barrier of O ions.

Alumina trihydrate can achieve the same IDC as alumina monohydrate obtained at high voltage when subjected to a lower voltage. However, increasing the electric field strength does not increase the IDC of alumina trihydrate; instead, it begins to decrease. This suggests that the presence of H ions in the hydrate does not consistently enhance ion diffusion and excessive H ions can consume a significant amount of electric field energy. Although the IDC of hydrogen was still increasing, the IDC of aluminium and oxygen ions in the oxide were decreasing. Therefore, only the hydrogen ions that have bonded with oxygen ions can facilitate ion migration in alumina. It is concluded that in the oxidation process of AAO, the role of hydrogen ion in hydration mainly includes: (a) providing an ion channel; (b) the drag effect in the migration process; (c) reducing the activation energy of ions.

**Figure 4:**
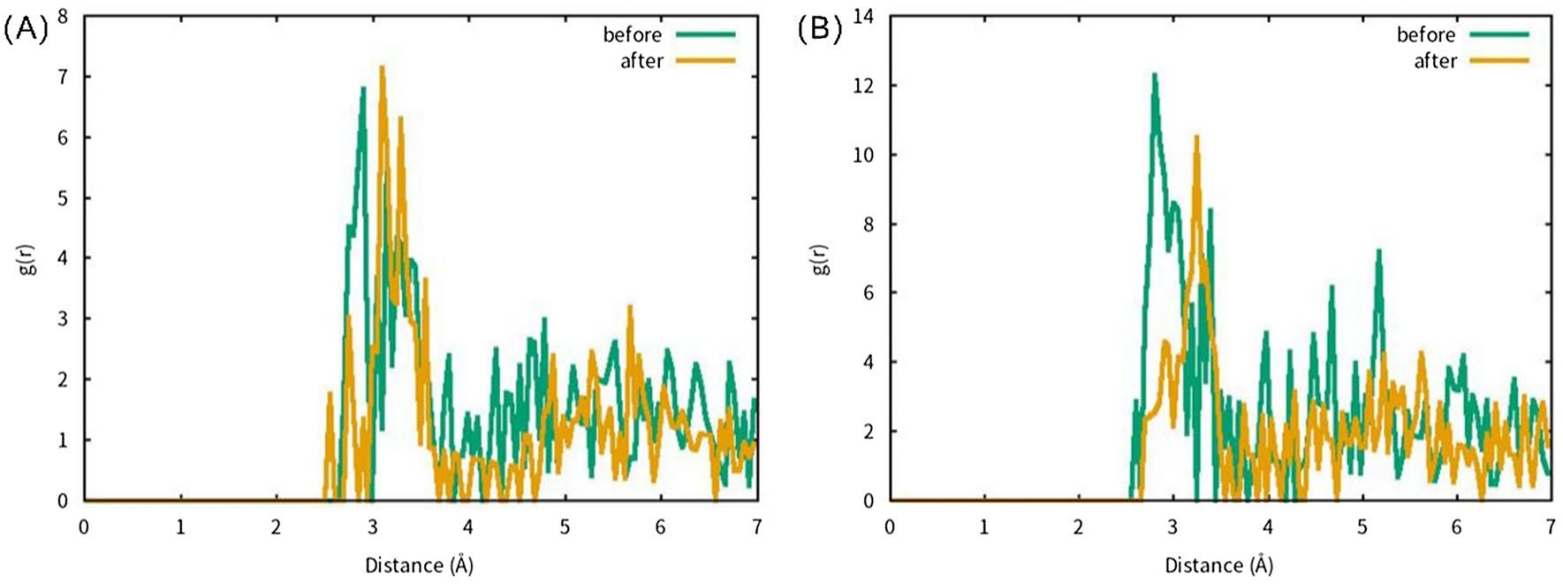
RDF of Al–Al in hydrated alumina, green line is no electric field, yellow line is under electric field. (**A**) 2 V/Å alumina monohydrate, (**B**) 0.5 V/Å alumina trihydrate.

The impact of hydrate content on the structure of alumina was observed in the radial distribution functions of alumina monohydrate at 2 V/Å and alumina trihydrate at 0.5 V/Å, as shown in Fig. 4. The electric field strength increased the spacing of Al ions, resulting in a change in the Al–Al distance of alumina monohydrate from 2.85 to 3.15 Å and that of alumina trihydrate from 2.75 to 3.25 Å. The structure of alumina was loosened due to hydration.

### Influence of electrolyte environment

The electrolyte plays a crucial role in the AAO process. Generally, anodic aluminium oxide forms a dense protective layer in neutral environments and a vertically porous structure with uniform distribution in acidic environments. In this article, H ions are used instead of O ions to give the system a positive charge and create an acidic environment suitable for simulating anodic oxidation. This ion replacement method is commonly used in simulating charged systems^29^. Table S2 displays the correlation among IDC, ion mobility of ions in hydrated alumina, and acidity under an electric field strength of 0.1 V/Å.

**Figure 5:**
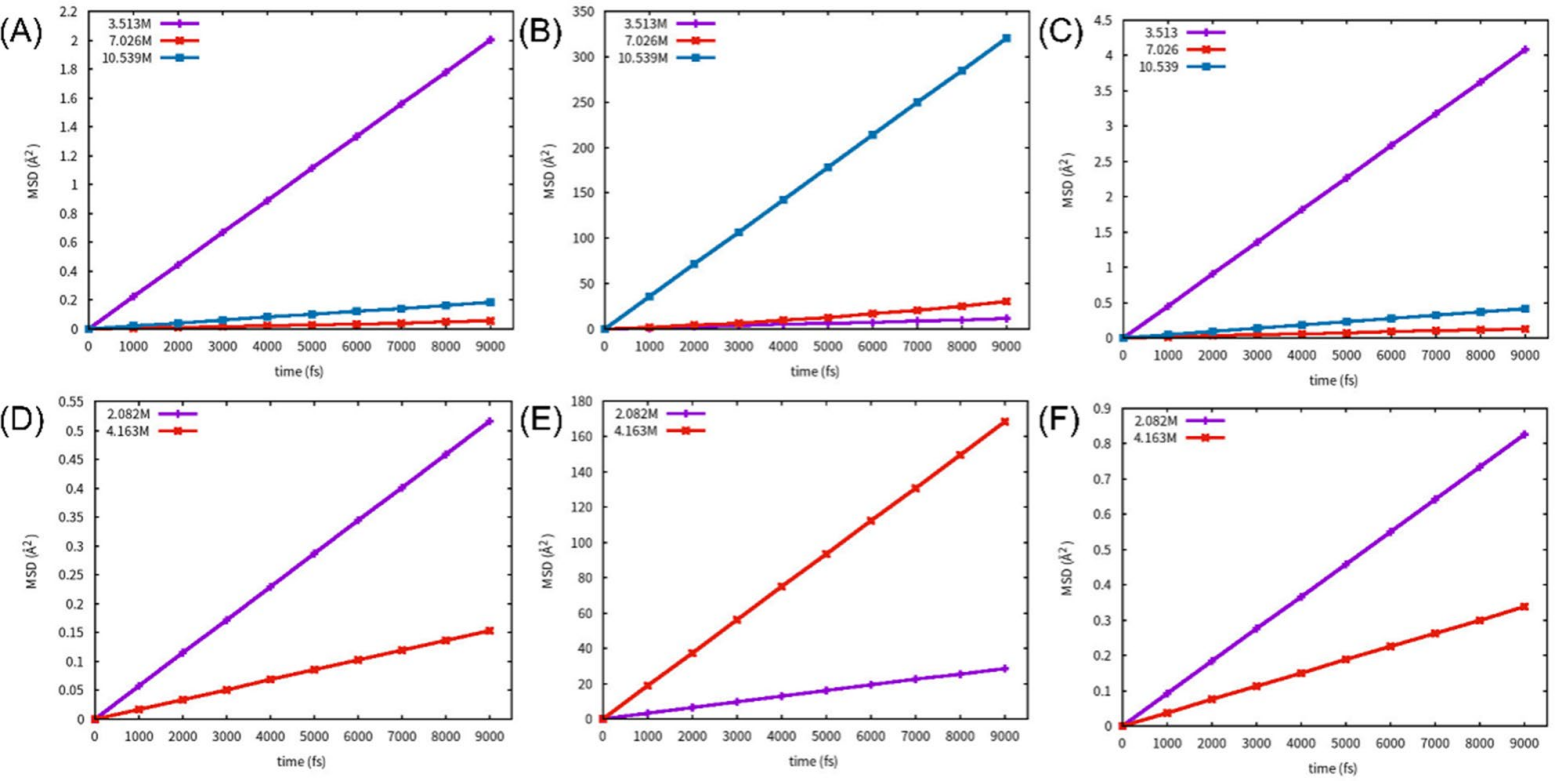
MSD under electric field of Al, H, O. (**A**) Al, (**B**) H, (**C**) O of alumina monohydrate under acid conditions. (**D**) Al, (**E**) H, (**F**) O of alumina trihydrate under acid conditions.

Figure 5 displays the MSD of hydrous alumina in an acidic environment. The IDC of H ions increased with increasing acidity, while the IDC of Al and O ions were significantly reduced in both alumina monohydrate and alumina trihydrate. Table S2 and the blue and yellow lines in Fig. S6 demonstrate that the IDC of H is much higher than that of Al and O. When combined with the electric field strength effect discussed in section “Influence of electric field strength”, the system would break down if the electric field were too strong. When the system malfunctioned, the charge (primarily in the form of H and electrons) quickly moved along the breakdown channel, resulting in a decrease in the IDC of the remaining ions instead of an increase. Trihydrate alumina, in comparison to monohydrate alumina, has a higher water content, lower density, and looser structure, resulting in a lower breakdown electric field. Therefore, at a concentration of 3.513 M H^+^, the alumina monohydrate remained intact, resulting in the highest diffusivity rate. Conversely, the alumina trihydrate was broken down, resulting in a much lower diffusivity rate than that of alumina monohydrate. Unless otherwise stated, the diffusivity rate refers to the IDC of Al ion with changes in H and O ion content. At an H ion concentration of 7.026 M, the diffusivity rate sharply decreased due to the breakdown of alumina monohydrate. The IDC of H was approximately 20 times higher than that of Al (or O) when the system broke down, as evidenced by voltage influence data. This information is a crucial reference for analysing the oxidation process of anodic aluminium oxide (AAO). It can be concluded that the IDC in the oxide layer is enhanced by the acidic environment, resulting in an increased oxidation rate. Higher acidity levels can cause the breakdown of hydrous compounds, which prevents the oxide thickness from increasing.

Existing experimental results suggest that the film growth rate increases with the acid concentration^34–37^. This is similar to our conclusion, as the growth rate is positively correlated with IDC and ion mobility.

## Conclusion

Molecular dynamics simulations were conducted using AIMD to investigate the behaviour of ions during the anodic alumina process in both anhydrous and hydrated amorphous alumina. The main conclusions are as follows:

1. As the electric field strength increases, the IDC of ions in alumina gradually increases. However, if the electric field strength is too strong, it can cause a breakdown current, which limits the diffusion rate of ions. In the simulation, the maximum Al IDC value of alumina monohydrate was found to be 21.8 μm^2^/ms/V. It is important to note that the content of oxide hydrate also affects the ion diffusion rate. Under low electric field intensity, alumina trihydrate demonstrates the highest IDC of aluminium at 16.7 μm^2^/ms/V with a 0.25 V/Å.
2. The IDC in anhydrous amorphous alumina was 1–2 orders of magnitude lower than that in hydrated oxide, even at high electric field strength. The reason for this phenomenon is the effect of H ions in hydrated alumina. Some of the ionised H ions exist in the form of “isolation” with a high diffusion rate and form conductive channels when the electric intensity is high. Another part of the H ions, which are still bound to the O ions, play a drag role in the diffusion of the O ions and can reduce the activation energy required for the movement of the O ions.
3. The diffusion rate of alumina is affected by the concentration of H+ ions in acidic electrolytes. The IDC of alumina increased as the acidity level rose in the acidic environment where the concentration of H ion was less than 3.52 M. However, if the acidity is too strong, the hydrous compound will be broken down by the electric field, and the IDC, except for that of the H ion, will not increase.

### Supplementary Information

Supplementary Information.

## Data Availability

The data involved in the article can be provided upon request (Email: 15703419053@163.com).
