# Supplementary material for: An ab initio molecular dynamics investigation of the behaviour of amorphous substances in anodic aluminium oxide under electric field
Source: Sci Rep. 2024 May 7;14:10454. doi: 10.1038/s41598-024-58975-y (PMC11076534; doi:10.1038/s41598-024-58975-y)
Supplement: Supplementary file 1 — Supplementary Information. [file 41598_2024_58975_MOESM1_ESM.docx]

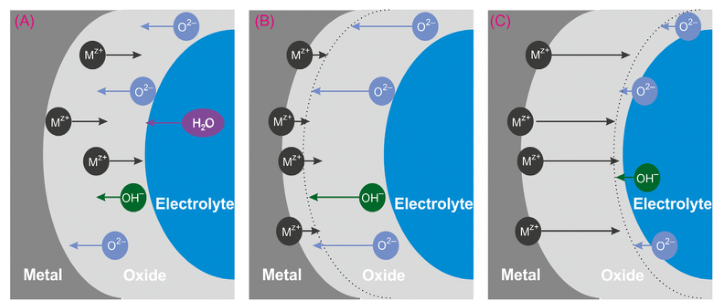


Figure S1 Metal oxide formation position diagram [1]: the different situations of Metal ions and oxygen ions diffusion rate. (A) not much different, (B) metal ions are slower, (C) metal ions are faster.


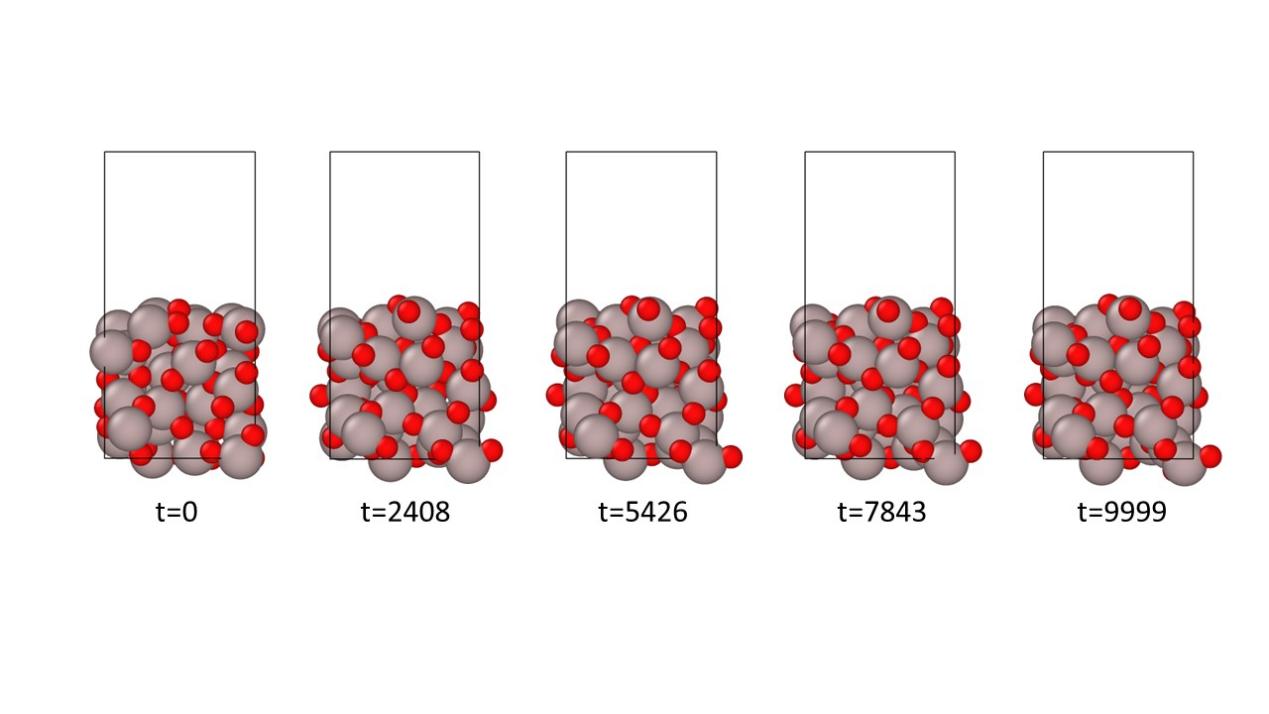


Figure S2 Amorphous alumina structure changes with time, time (t) unit is fs, electric field strength is 2 V/Å, red ball is O, gray ball is Al.


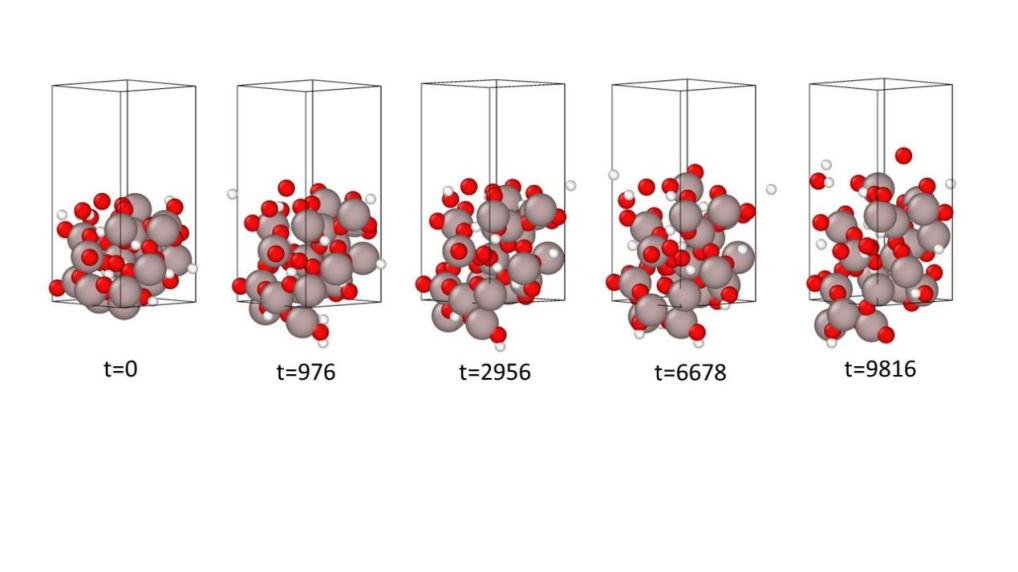


Figure S3 Alumina monohydrate structure changes with time, time (t) unit is fs, electric field strength is 2 V/Å, red balls are O atoms, gray balls are Al, white balls are H.


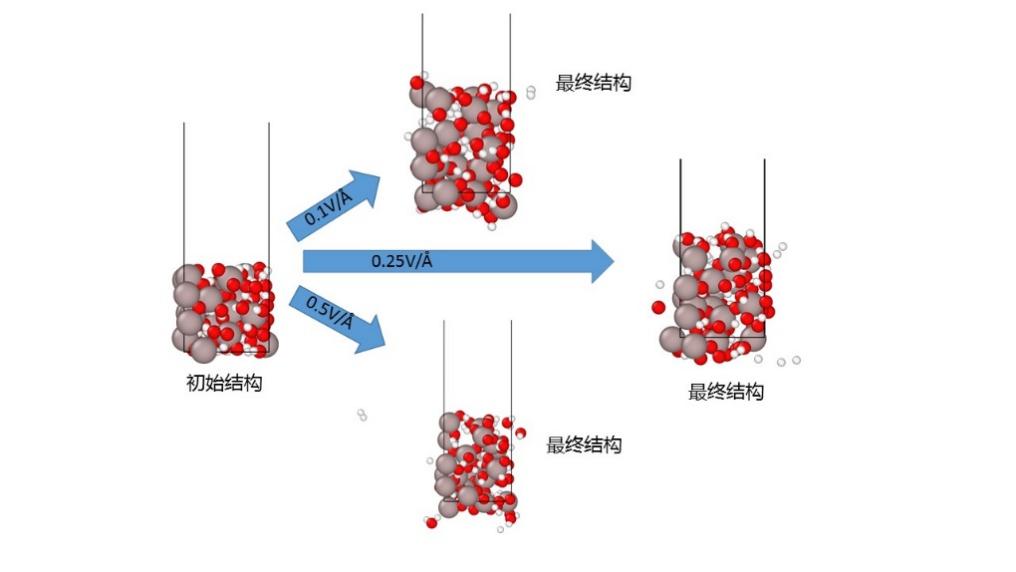


Final structure

Initial structure

Final structure

Final structure

Figure S4 Alumina trihydrate structure changes with time, red balls are O atoms, gray balls are Al, white balls are H.


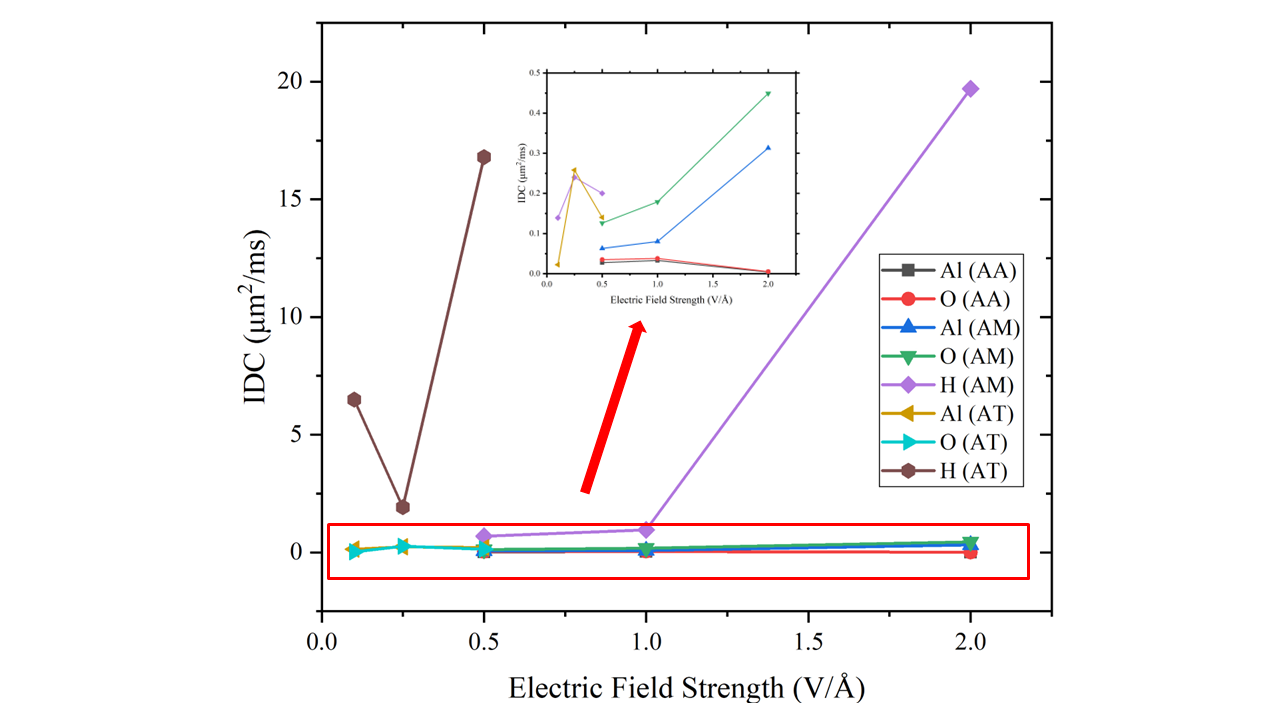


Figure S5 The IDC (μm^2^/ms) of ions Al, O and H in three models under different electric field strength. The illustration is an enlargement of the content within the red box. The AA in the legend is the abbreviation for Amorphous Alumina, the AM is the abbreviation for Alumina Monohydrate, and the AT is the abbreviation for Alumina Trihydrate.


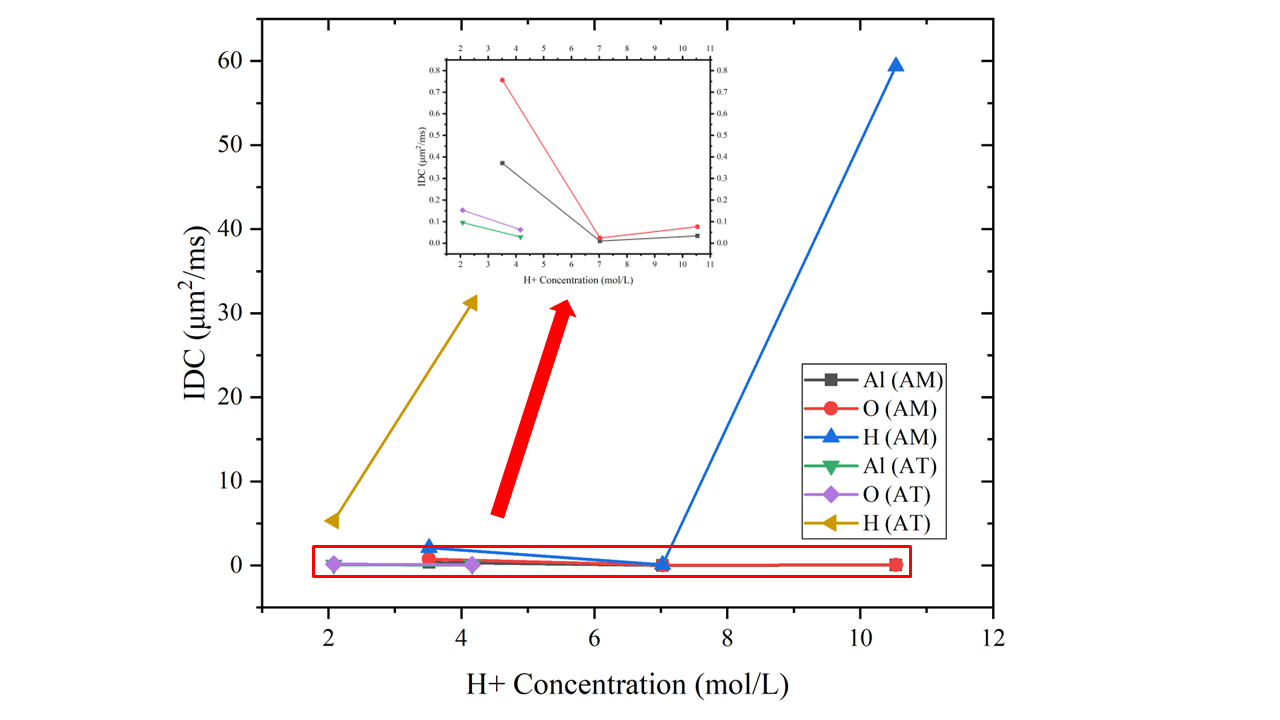


Figure S6 The IDC (μm^2^/ms) of Al, O, H under different acid condition with an electric field strength 0.1 V/Å. The illustration is an enlargement of the content within the red box. The AM in the legend is the abbreviation for Alumina Monohydrate, and the AT is the abbreviation for Alumina Trihydrate.

Table S2 The IDC and ion mobility of Al, O, H under different acid condition with an electric field strength 0.1 V/Å. Diffusion Coefficient unit: μm^2^/ms, Ionic Mobility unit: μm^2^/ms/V.

| IDC | (Electric Field Strength 0.1 V/Å) | Alumina Monohydrate | | | Alumina Trihydrate | |
| --- | --- | --- | --- | --- | --- | --- |
|  | H^+^ concentration (mol/L) | 3.513 | 7.026 | 10.539 | 2.082 | 4.163 |
|  | Ions Al | 0.371 | 0.00998 | 0.034 | 0.0956 | 0.0285 |
|  | Ions O | 0.756 | 0.0242 | 0.0762 | 0.153 | 0.0626 |
|  | Ions H | 2.10 | 0.0772 | 59.4 | 5.32 | 31.2 |
| Ion Mobility | (Electric Field Strength 0.1 V/Å) | Alumina Monohydrate | | | Alumina Trihydrate | |
|  | H^+^ concentration (mol/L) | 3.513 | 7.026 | 10.539 | 2.082 | 4.163 |
|  | Ions Al | 25.8 | 0.695 | 2.37 | 6.65 | 1.98 |
|  | Ions O | -35.1 | -1.12 | -3.54 | -7.10 | -2.90 |
|  | Ions H | 48.7 | 179 | 1380 | 124 | 724 |

Table S1 The IDC (μm^2^/ms) and ion mobility (μm^2^/ms/V) of ions Al, O and H in three models under different electric field strength.

| IDC |  | Electric Field Strength | 0.5 V/Å | 1 V/Å | 2 V/Å |
| --- | --- | --- | --- | --- | --- |
|  | Amorphous  Alumina | Ions Al | 0.0274 | 0.0328 | 0.00396 |
|  |  | Ions O | 0.0349 | 0.0379 | 0.00504 |
|  |  | Electric Field Strength | 0.5 V/Å | 1 V/Å | 2 V/Å |
|  | Alumina  Monohydrate | Ions Al | 0.0629 | 0.0801 | 0.313 |
|  |  | Ions O | 0.126 | 0.179 | 0.449 |
|  |  | Ions H | 0.688 | 0.962 | 19.7 |
|  |  | Electric Field Strength | 0.1 V/Å | 0.25 V/Å | 0.5 V/Å |
|  | Alumina  Trihydrate | Ions Al | 0.139 | 0.240 | 0.20 |
|  |  | Ions O | 0.0224 | 0.258 | 0.14 |
|  |  | Ions H | 6.49 | 1.92 | 16.8 |
| Ion  Mobility |  | Electric Field Strength | 0.5 V/Å | 1 V/Å | 2 V/Å |
|  | Amorphous  Alumina | Ions Al | 1.91 | 2.29 | 0.276 |
|  |  | Ions O | −1.62 | −1.76 | −0.234 |
|  |  | Electric Field Strength | 0.5 V/Å | 1 V/Å | 2 V/Å |
|  | Alumina  Monohydrate | Ions Al | 4.38 | 5.58 | 21.8 |
|  |  | Ions O | −5.83 | −8.30 | −20.8 |
|  |  | Ions H | 16 | 22.3 | 458 |
|  |  | Electric Field Strength | 0.1 V/Å | 0.25 V/Å | 0.5 V/Å |
|  | Alumina  Trihydrate | Ions Al | 9.66 | 16.7 | 13.9 |
|  |  | Ions O | −1.04 | −12 | −6.48 |
|  |  | Ions H | 151 | 44.6 | 155 |

**Reference**

[1] Sulka, Grzegorz D. "Introduction to anodization of metals." Nanostructured Anodic Metal Oxides. 1-34 (Elsevier, 2020).
